# Differing Endoplasmic Reticulum Stress Response to Excess Lipogenesis versus Lipid Oversupply in Relation to Hepatic Steatosis and Insulin Resistance

**DOI:** 10.1371/journal.pone.0030816

**Published:** 2012-02-15

**Authors:** Lu-Ping Ren, Stanley M. H. Chan, Xiao-Yi Zeng, D. Ross Laybutt, Tristan J. Iseli, Ruo-Qiong Sun, Edward W. Kraegen, Gregory J. Cooney, Nigel Turner, Ji-Ming Ye

**Affiliations:** 1 Molecular Pharmacology for Diabetes Group, Health Innovations Research Institute and School of Health Sciences, RMIT University, Melbourne, Victoria, Australia; 2 Diabetes and Obesity Research Program, Garvan Institute of Medical Research, Sydney, New South Wales, Australia; 3 Faculty of Medicine, University of New South Wales, Sydney, New South Wales, Australia; University of Geneva, Switzerland

## Abstract

Mitochondrial dysfunction and endoplasmic reticulum (ER) stress have been implicated in hepatic steatosis and insulin resistance. The present study investigated their roles in the development of hepatic steatosis and insulin resistance during *de novo* lipogenesis (DNL) compared to extrahepatic lipid oversupply. Male C57BL/6J mice were fed either a high fructose (HFru) or high fat (HFat) diet to induce DNL or lipid oversupply in/to the liver. Both HFru and HFat feeding increased hepatic triglyceride within 3 days (by 3.5 and 2.4 fold) and the steatosis remained persistent from 1 week onwards (p<0.01 vs Con). Glucose intolerance (iAUC increased by ∼60%) and blunted insulin-stimulated hepatic Akt and GSK3β phosphorylation (∼40–60%) were found in both feeding conditions (p<0.01 vs Con, assessed after 1 week). No impairment of mitochondrial function was found (oxidation capacity, expression of PGC1α, CPT1, respiratory complexes, enzymatic activity of citrate synthase & β-HAD). As expected, DNL was increased (∼60%) in HFru-fed mice and decreased (32%) in HFat-fed mice (all p<0.05). Interestingly, associated with the upregulated lipogenic enzymes (ACC, FAS and SCD1), two (PERK/eIF2α and IRE1/XBP1) of three ER stress pathways were significantly activated in HFru-fed mice. However, no significant ER stress was observed in HFat-fed mice during the development of hepatic steatosis. Our findings indicate that HFru and HFat diets can result in hepatic steatosis and insulin resistance without obvious mitochondrial defects via different lipid metabolic pathways. The fact that ER stress is apparent only with HFru feeding suggests that ER stress is involved in DNL *per se* rather than resulting from hepatic steatosis or insulin resistance.

## Introduction

Non-alcoholic fatty liver disease (NAFLD) affects approximately 10–20% of the population and is a hepatic manifestation of the metabolic syndrome which includes insulin resistance, obesity and type 2 diabetes [1], [2]. NAFLD defines a spectrum of liver abnormalities from benign simple non-alcoholic fatty liver (NAFL or steatosis) to steatohepatitis (NASH) which is associated with inflammation and liver damage [2]. Although the causal relationship between hepatic steatosis and insulin resistance is a matter of debate, NAFL is believed to be a prerequisite for NASH [3].

The effect of dietary fructose and fat on the development of NAFL and insulin resistance has attracted much attention due to their overconsumption in the modern society [4], [5]. A number of studies including our own have revealed the critical role of active lipid metabolites such as long chain fatty acyl-CoAs, diacylglycerol and ceramide in generating insulin resistance in muscle and liver [6], [7]. As well as being an important site of fatty acid oxidation, the liver is also a major organ for *de novo* lipogenesis and its insulin sensitivity appears to be more vulnerable to the insult of lipid accumulation compared to muscle [8].

It has been suggested that defects in mitochondrial substrate oxidation would cause lipid accumulation [9] and thus insulin resistance. In the liver, partial deletion of a key mitochondrial protein for β-oxidation causes hepatic steatosis and insulin resistance [10]. Furthermore, mitochondrial dysfunction has been demonstrated to occur prior to the appearance of hepatic steatosis and insulin resistance [11]. While these findings highlight the potential role of mitochondrial dysfunction in NAFL, it is not known whether this is a primary cause of hepatic steatosis and insulin resistance or arises as a secondary defect [12].

Recently, endoplasmic reticulum (ER) stress has been proposed as a key intersection of lipogenesis, inflammation and insulin resistance in the liver [13], [14]. ER stress has been reported to promote a JNK-dependent serine phosphorylation of IRS-1 and inhibit insulin action in cultured liver cells [15], [16]. Activation of key ER stress signalling molecules has also been shown to enhance lipogenesis, contributing to hepatic steatosis and insulin resistance [17]. However, it is not known whether ER stress is also associated with an increase in DNL or lipid influx, as the majority of the existing data was derived from genetically obese or prolonged chronic high fat feeding models [13], [15], [16], [18].

As high-fat (HFat) and high-fructose (HFru) diets are known to cause hepatic steatosis by increased extrahepatic lipid supply and hepatic DNL, respectively [19], [20], the present study aimed to examine the role of mitochondrial dysfunction and ER stress in the development of hepatic steatosis and insulin resistance induced by these two distinct lipid metabolic pathways. Our findings show that the development of hepatic steatosis and insulin resistance resulting from excessive DNL is closely associated with ER stress but not mitochondrial dysfunction. In contrast, lipid oversupply induced steatosis and insulin resistance occurred along with JNK activation but without ER stress. The present study suggests a divergence in ER stress pathways between intrahepatic DNL and extrahepatic lipid supply on the initiation of hepatic steatosis and insulin resistance.

## Materials and Methods

### Animals

Male C57BL/6J mice (14 weeks) from the Animal Resources Centre (Perth, Australia) were kept at 22±1°C on a 12-h light/dark cycle. After 1 week of acclimatization, mice were fed *ad libitum* for up to 8 weeks with either a chow (CH), HFru, or HFat diet. CH diet consisted of 70% calories from starch, ∼10% calories from fat, and ∼20% calories from protein (Gordon's Specialty Stock Feeds, Yanderra, Australia), HFru diet (35% fructose, 35% starch, ∼10% fat and ∼20% protein), a HFat diet (60% from saturated fat, 20% protein, and ∼20% carbohydrate). The detailed recipes for HFru and HFat diets are described in our previous studies [19], [21]. All experiments were approved by the Animal Ethics Committees of the Garvan Institute (#0847) and RMIT University (#1012).

Body weight and food intake were measured twice weekly. The whole body metabolic rate was assessed at 22°C using an indirect calorimeter (Oxymax, Columbus Instruments, OH, USA) as described previously [22], between 4–7 days after the commencement of the HFru or HFat feeding. Following 5–7 hours of fasting, mice were culled and tissues of interest were collected and freeze-clamped immediately. Epididymal fat mass was weighed using an analytical balance. Liver triglycerides were extracted by the method of Folch [6], [23] and determined by a Peridochrom Triglyceride GPO-PAP kit (Roche Diagnostics, Australia). In separate experiments, glucose tolerance tests (GTT; 3 g glucose/kg BW, *i.p.*) were performed using a glucometer (AccuCheck II; Roche, New South Wales, Castle Hill, Australia). For the assessment of insulin signaling in the liver, the mice were injected with insulin (2 U/kg BW, *i.p.*) along with glucose (3 g/kg BW) to avoid hypoglycemia 40 min before tissues were collected. DNL was determined by measuring the incorporation of [^3^H]-H_2_O into triglyceride in the liver as described previously [24]. Briefly, mice were injected with [^3^H]-H_2_O (20 µCi/g BW, *i.p.*). Blood sample and liver samples were collected at 90 min for the measurement of radioactivity in plasma and liver triglyceride using a β-scintillation counter.

### Mitochondrion-dependent oxidation of substrates

Palmitate and glutamate oxidations were measured in liver homogenates using methods described previously [12] with modifications. Briefly, approximately 30 mg of fresh liver was homogenized in an ice-cold Tris buffer containing (in mM) 250 sucrose, 10 Tris-HCl and 1 EDTA, pH 7.4. The liver homogenate (50 µl) was incubated with a 450 µl reaction mixture (pH 7.4). Final concentrations of the reaction mixture were (in mM): 100 sucrose, 80 KCl, 10 Tris-HCl, 5 KH_2_PO_4_, 1 MgCl_2_, 2 malate, 2 ATP, 1 dithiothreitol, 0.2 EDTA, and 0.3% fatty acid–free BSA. Substrates (in mM) were 0.2 [1-^14^C]-palmitate (0.5 µCi) plus 2 l-carnitine and 0.05 coenzyme A or 10 [1-^14^C]-glutamate (0.1 µCi). After 90 min of incubation at 30°C, the reaction was stopped by 0.1 ml of ice-cold 1 M perchloric acid. CO_2_ produced during the incubation was captured in 0.1 ml of 1 M NaOH. For palmitate oxidation, ^14^C counts present in the acid-soluble fraction were also measured and combined with the CO_2_ values to give the total palmitate oxidation rate.

### Citrate synthase and β3 – hydroxyacyl– CoA dehydrogenase (β-HAD) activity

Approximately 20 mg of frozen liver tissue was homogenized in 175 mM KCl and 1.98 mM EDTA containing buffer (pH 7.4) with a glass homogenizer before subjected to three freeze-thaw cycles. Citrate synthase and β-HAD activities were determined as described previously [25], [26], using a Flexstation 3 plate reader (Molecular Devices, Sunnyvale, CA). Enzyme activities are presented as units per gram wet weight, where units are defined as micromoles per minute.

### NADPH oxidase activity and glutathione (GSH) content

NADPH oxidase-driven superoxide production in the frozen liver tissue was determined using lucigenin-enhanced chemiluminescence. Approximately 10 mg of tissue was pre-incubated in Krebs–HEPES buffer containing diethylthiocarbamic acid (1 mM) at 37°C for 45 min to inactivate superoxide dismutase. NADPH (100 mM), a substrate for NADPH oxidase was supplemented either alone or in the presence of diphenylene iodonium (5 mM), which is a flavoprotein inhibitor that inhibits NADPH oxidase. Follow by 300 uL of Krebs-HEPES buffer containing lucigenin (5 mM) and the appropriate sample were placed into a 96-well Optiplate, and superoxide production was measured and quantified as previously described [27]. Intracellular GSH content was assessed in de-proteinated whole-liver lysate using the glutathione assay kit (Cayman Chemical Co., Ann Arbor, MI) according to manufacturer's instruction. Briefly, 10 mg of frozen tissue was homogenized in the MES buffer provided. The homogenates were then de-proteination using metaphosphoric acid and triethanolamine, the supernatant was then collected for the analysis of intracellular GSH content. Data is expressed as micro molar of GSH per mg of wet tissue weight.

### Western blotting

Liver samples were homogenized in ice-cold lysis buffer at pH 7.5 containing (in mM): 50 Tris, 150 NaCl, 1% Triton X-100, 10 NaP, 100 NaF, 2 Na3VO4, 1 EDTA, 1 EGTA and 10% glycerol supplemented with protease inhibitor cocktail tablets (Roche Diagnostics Pty Ltd, Australia) and DL-dithiothreitol. Protein samples were then denatured in SDS sample buffer (125 mM Tris-HCl, pH 6.8, 50% glycerol, 2% SDS, 5% β-mercaptoethanol and 0.01% bromophenol blue). The insulin signal transduction was assessed by total- and phospho (Ser473)- Akt, total- and phospho (Ser219)- glycogen synthase kinase 3β (GSK-3β, Cell Signaling, USA). Key lipogenic enzymes were by Western blotting using specific antibodies including acetyl-CoA carboxylase (ACC, Upstate, USA), fatty acid synthase (FAS, Abcam, USA), stearoyl-CoA desaturase 1 (SCD-1, Cell signaling, USA). Mitochondrial metabolic capacity: proliferator–activated receptor co-activator 1α (PGC-1α, Chemicon International, Temecula, USA), voltage dependent anion channel (VDAC, Cell signaling, USA), carnitine palmitoyltransferase-1 (CPT-1, Santa Cruz, USA), cytochrome oxidase subunit 1(COX-1, Abcam, USA) and an antibody cocktail that recognizes several subunits of the mitochondrial respiratory chain (MS601; Mitosciences, Eugene, USA). ER stress: total- and phospho (Thr980)- pancreatic ER kinase (p-PERK, Cell signaling, USA), total- and phospho (Ser724)- inositol-requiring kinase 1 (IRE1, Abcam, USA), ATF-6 α, total- and phospho (Ser51)- eukaryotic translation initiation factor 2α (eIF2α, Santa Cruz, USA). Stress-activated kinases: total- and phospho (Thr183/Tyr185)-c-Jun N-terminal kinase (JNK), total- and phospho (Ser176/180)- IκB kinase α/β (total-IKK α/β) and IκBα (Cell Signaling, USA). Immunolabeled bands were quantified by densitometry and representative blots are shown.

### Analysis of gene expression

Total RNA was extracted from liver tissue using TRIZOL® (Invitrogen, USA) according to manufacturer's instructions. Reverse transcription was carried out using 0.2 µg of RNA using the High Capacity cDNA Reverse Transcription Kit (Applied Biosystems, USA). Real time PCR was carried out using the IQ SYBR Green Supermix (2X) (Biorad Laboratories Inc, USA) for murine sterol regulatory element-binding protein-1c (SREBP-1c) and carbohydrate responsive element binding protein (ChREBP). Primer sequences are described in Table S1. The gene expression from each sample was analysed in duplicates and normalized against the housekeeper 18S. All reactions were performed on the iQ™5 Real Time PCR Detection System (Biorad Laboratories Inc, USA). The results are expressed as relative gene expression using the ΔCt method.

### XBP1 mRNA cleavage by IRE1

Total RNA extracted was PCR using the specific primer set for mouse X-box binding protein (XBP1; Table S1) which amplifies a 601-bp cDNA product encompassing the IRE1 cleavage sites. This fragment was further digested by *Pst*I to reveal a restriction site that is lost after IRE1-mediated cleavage and splicing of the mRNA. The cDNA fragments were resolved on 2% agarose gels and analysed by FluorChem® Imaging System (Alpha Innotech, USA).

### Statistical analyses

Data are presented as means ± SE. One-way analysis of variance was used for comparison of relevant groups. When significant differences were found, the Tukey-Kramer multiple comparisons test was applied. Differences at *p*<0.05 were considered to be statistically significant.

## Results

### Both HFru and HFat feeding led to hepatic steatosis

**Figure 1:**
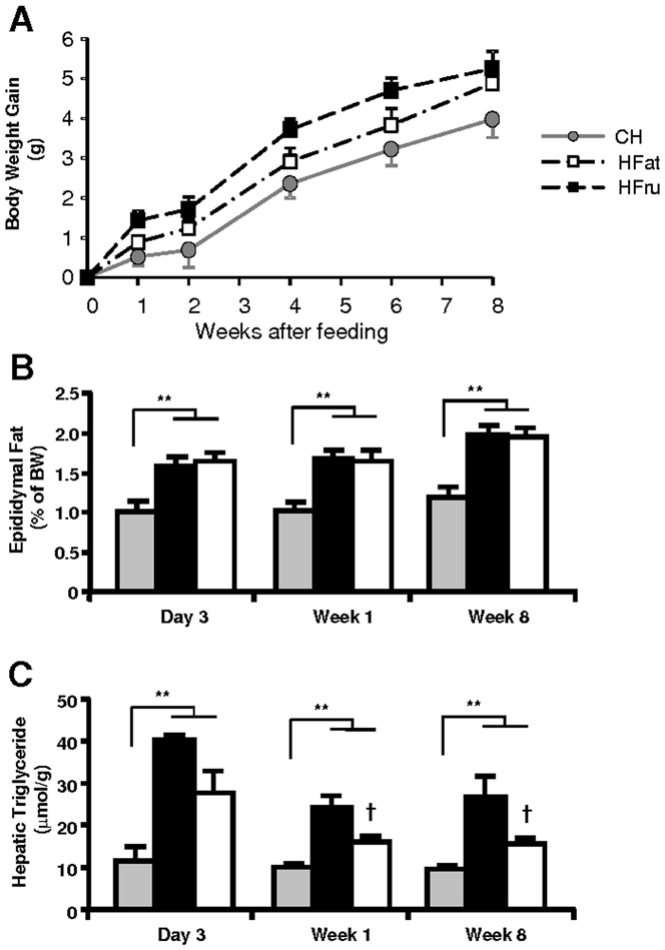
Effects on HFru and HFat feeding on weight gain, adiposity and liver triglyceride. Male C57BL/6J mice (13–16 weeks old) were fed a high-fructose (HFru) or a high-fat (HFat) diet *ad libitum* as compared to a standard laboratory chow diet (CH). The body weight was similar among the three groups at the start of the feeding intervention (23.6±0.3, 23.4±0.5 and 23.7±0.5 g). (**A**) Body weight gain over the baseline. (**B**) Weight of epididymal fat depots. (**C**) Liver triglyceride content. Liver samples were freeze-clamped for the measurement of hepatic triglyceride content after 5–7 hour of fasting. Data are mean ± SE of 8–13 mice per group. ** *p*<0.01; † *p*<0.05, †† *p*<0.01 vs HFru.

Both HFru and HFat feeding tended to cause slightly more weight gain starting at week 1 (Figure 1A) and significantly increased adiposity (60% increase in epididymal fat mass, *p*<0.05), which occurred as early as 3 days (Figure 1B). The caloric intake was greater in both HFru (by 25% vs CH, *p*<0.01) and HFat group (by 30% vs CH, *p*<0.01) in the first week which was gradually stabilized to be ∼10–12% in both groups over 8 weeks of feeding (Table 1). The basal metabolic rate was enhanced in mice fed a HFru or HFat diet (10% and 12% increase in VO_2_, respectively, *p*<0.01) compared to CH-fed mice (Table 1). Basal plasma glucose was slightly elevated after 8 weeks of HFru or HFat feeding while plasma levels of insulin and triglyceride were unchanged (Table 1).

**Table 1:** Basal metabolic parameters of HFru and HFat-fed mice.

	CH	HFru	HFat
Body mass (g)			
Week 1	24.1±0.4	24.9±0.5	24.5±0.5
Week 8	27.8±0.6	28.7±0.6	28.0±0.8
Caloric intake (Kcal/kg.day)			
Week 1	430±7	539±16**	572±23**
Week 8	443±11	497±24*	498±4**
VO_2_ (ml/kg/h)			
Week 1	3368±27	3733±35**	3968±38**
Blood glucose (mM)			
Week 1	6.2±0.2	6.2±0.3	6.6±0.3
Week 8	6.6±0.2	7.8±0.4*	8.2±0.2**
Plasma insulin (nmol/L)			
Week 1	ND	ND	ND
Week 8	0.22±0.02	0.25±0.03	0.19±0.02
Plasma triglyceride (mM)			
Week 1	0.99±0.02	1.11±0.06	1.11±0.06
Week 8	1.15±0.13	0.78±0.06	1.03±0.07

ND, not determined. Data are means ± SE of 10–15 mice per group.

*p<0.05,

**p<0.01 vs CH-fed mice.

As expected, the hepatic triglyceride content was readily augmented (after 3 days) in response to HFru or HFat feeding (*p*<0.01; Figure 1C) indicating the development of steatosis. Interestingly, HFru feeding resulted in a more pronounced triglyceride accumulation than that of HFat feeding during the 8 weeks of the study. As elevated liver triglyceride content was found to remain similarly elevated (HFru 2.8 fold, HFat 1.5 fold, *p*<*0.01*) from week 1 onwards (Figure 1C), our subsequent studies focused on the one week model to investigate the mechanism involved in the development of hepatic steatosis and insulin resistance.

### Both HFru and HFat feeding resulted in glucose intolerance and impairment in hepatic insulin signal transduction

**Figure 2:**
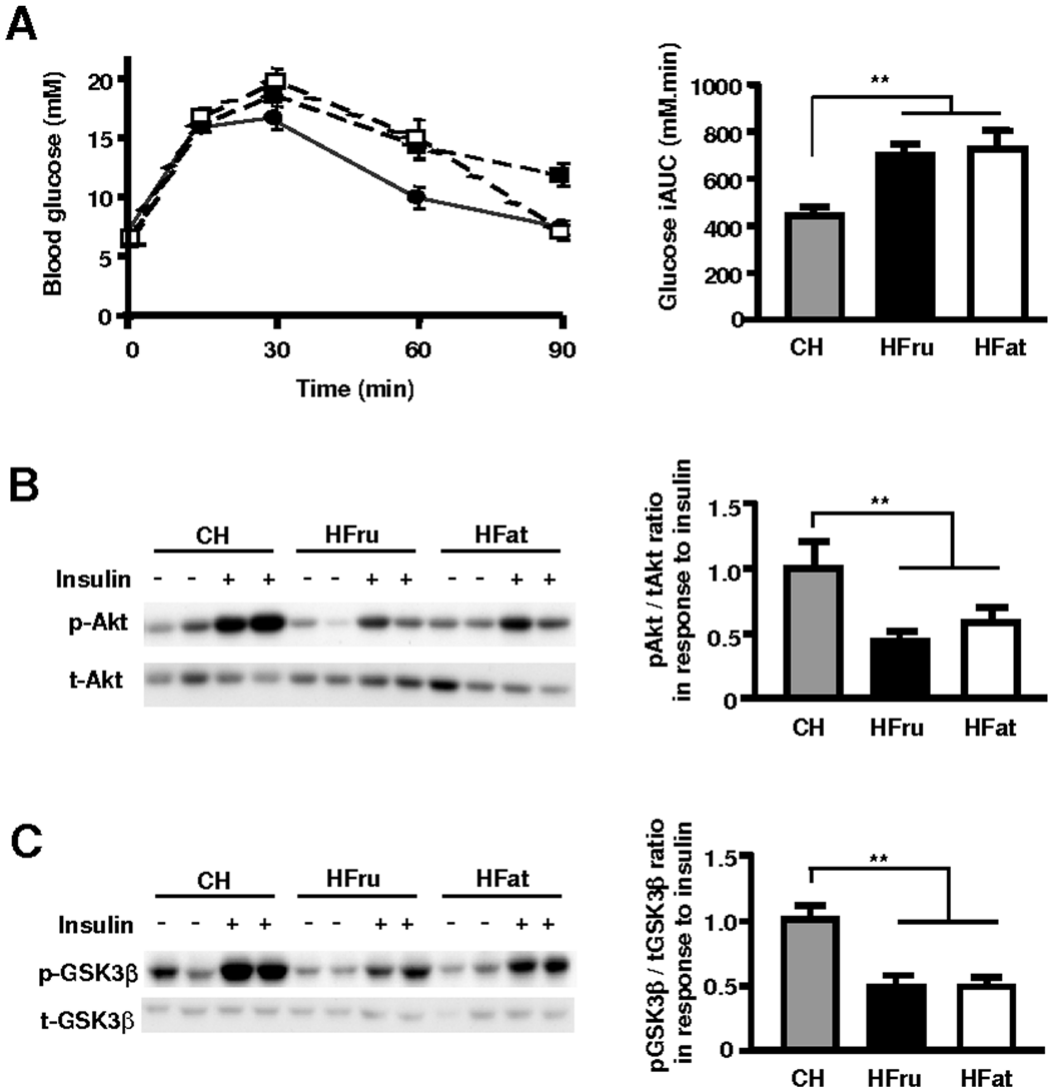
Effects of HFru and HFat feeding on glucose tolerance and hepatic insulin signal transduction. The experiments were performed after one week of high fructose (HFru, ▪), high fat (HFat, □) or chow (CH, •) feeding. (**A**) Glucose tolerance test (GTT) was performed with an injection of glucose (3 g/kg, *ip*) after 5–7 hours of fasting. Data are mean ± SE, 6–10 mice per group. iAUC, incremental area under the curve for blood glucose level. Insulin signal transduction was assessed by immunoblotting of phosphorylated-Akt (Ser473) (**B**) and -GSK3β (Ser219) (**C**) in the liver in response to a bolus of insulin stimulation (2 U/kg, *ip*). Each lane represents a single mouse. Densitometry data are mean ± SE of 6 mice per group. ** *p*<0.01.

HFru and HFat-fed mice displayed impaired glucose tolerance, as reflected by a 58% and 64% greater incremental area under the curve (iAUC) of blood glucose, respectively (*p*<0.01 vs CH-fed mice; Figure 2A). To assess the effect of the diets on hepatic insulin signal transduction, we examined the phosphorylation of the key signaling proteins, Akt and GSK-3β, in response to insulin stimulation. As expected, insulin-stimulated Akt phosphorylation was blunted in both HFru (57%, *p*<0.01) and HFat-fed (42%, *p*<0.01) compared with CH-fed mice (Figure 2B). Consistent with these changes, the insulin-stimulated phosphorylation of GSK-3β was also reduced (53% in HFru and 52% in HFat mice, *p*<0.01; Figure 2C). These results indicate decreased hepatic insulin sensitivity in both HFru and HFat-fed mice.

### HFru and HFat feeding exerted opposite effects on hepatic *de novo* lipogenesis

**Figure 3:**
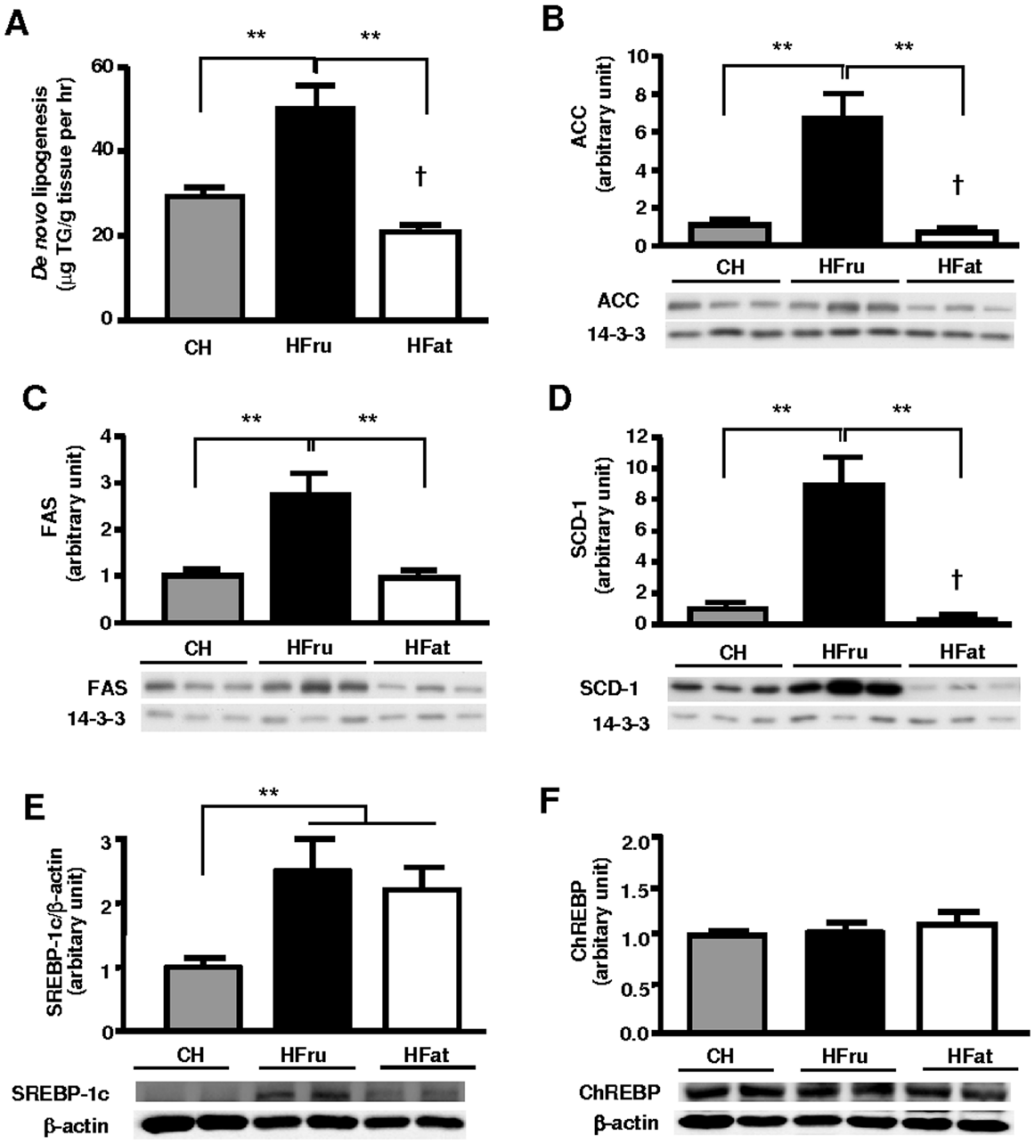
Effects of HFru and HFat on hepatic DNL, lipogenic protein levels and protein expression of SREBP-1c and ChREBP. After one week of HFru or HFat feeding, (**A**) hepatic DNL was measured by the incorporation of [^3^H]-H_2_O into hepatic triglyceride (n = 8/group). Protein levels of lipogenic enzymes ACC (**B**), FAS (**C**) and SCD-1 (**D**) were determined by immunoblots. Each lane represents a single mouse. Densitometry data are mean ± SE of 6 mice per group. Protein expressions of the lipogenic transcription factors SREBP-1c (**A**) and ChREBP (**B**) were determined by western blotting. Data are mean ± SE relative to CH-fed mice of 6 mice per group. * *p*<0.05, ** *p*<0.01. † *p*<0.05 vs CH.

As hepatic steatosis can result from an increase in either *de novo* lipogenesis (DNL) or influx of fatty acids, we measured the rate of incorporation of [^3^H]-H_2_O into triglyceride to determine whether DNL was altered by HFru or HFat feeding. As expected, HFru feeding resulted in an increased rate of DNL (63%, *p*<0.01) while HFat feeding decreased DNL (32%, *p*<0.05; Figure 3A). In agreement with these metabolic changes, HFru-fed mice exhibited dramatic increases in the protein expression of ACC (by 6.4-fold), FAS (by 2.7-fold) and SCD-1 (by 8.0-fold) (*p*<0.01; Figure 3B, C and D). In contrast, HFat feeding reduced the protein expression of ACC (by 45%) and SCD-1 (by 78%) (both *p*<0.05), but not FAS. DNL is believed to be under the regulation of two master transcription factors: SREBP-1c and ChREBP [1], [2], [28], [29], [30]. We found a significant increase of SREBP-1c protein expression in both HFru- (2.3 fold) and HFat-feeding (2 fold) (*p*<0.01 vs Ch; Figure 3E), whereas the protein expression of ChREBP remained unaltered (Figure 3F). Interestingly, HFru feeding significantly increased the mRNA expression of both SREBP-1c (3.3 fold, *p*<0.01) and ChREBP (44%, *p*<0.05; Figure S1A and B), while HFat feeding also resulted in a 50% induction of SREBP-1c mRNA expression (*p*<0.01). These results further demonstrated the effects of HFru and HFat feeding on different lipid metabolic pathways.

### HFru and HFat feeding did not alter mitochondrial metabolism in the liver

**Figure 4:**
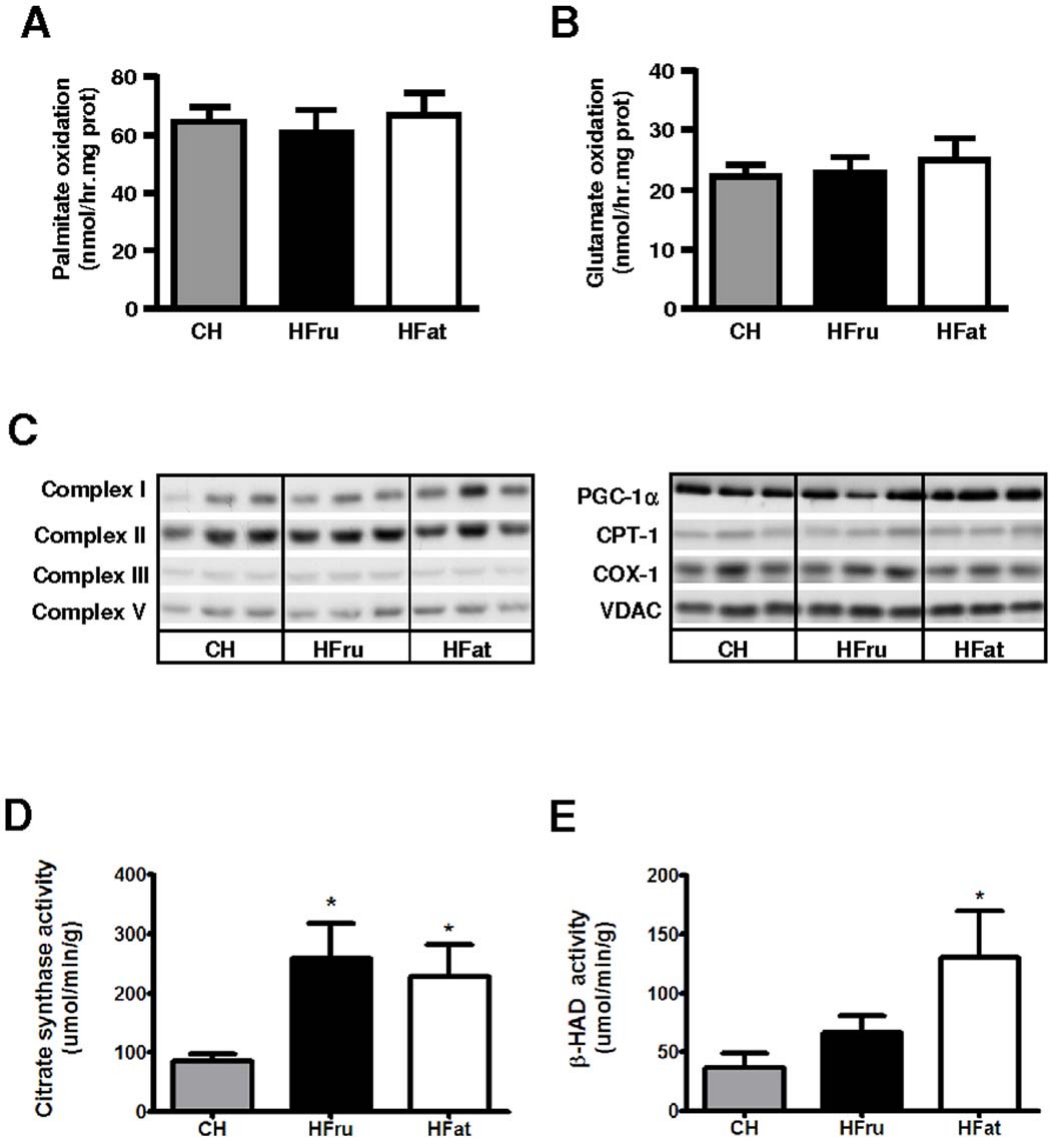
Effects of HFru and HFat feeding on mitochondrial oxidation, key protein expressions and enzymatic activities. After one week of HFru or HFat feeding, the rates of hepatic oxidation of palmitate (**A**) and glutamate (**B**) were measured in liver homogenates using [1-^14^C]-palmitate or [1-^14^C]-glutamate as substrates. Data are mean ± SE from 6 mice per group. (**C**) Levels of key proteins for mitochondrial oxidation, biogenesis and numbers were measured by immunoblots. Each lane represents a single mouse, 6 mice per group. The specific activity of citrate synthase (**D**) and β-HAD (**E**), data are mean ± SE from 6 to 8 mice per group. * *p*<0.05 vs CH.

Given the important role of the mitochondria in substrate utilisation, we next sought to examine whether mitochondrial functions were altered by HFru feeding. Measurement of mitochondrial oxidation capacity from the liver homogenates using palmitate and glutamate as substrates revealed no difference compared to the CH group (Figure 4A and B). Consistent with this, we also found no defects in the expression level of key proteins in the respiratory chain (complex I–III and V; Figure 4C). Neither the key regulators of mitochondrial biogenesis (PGC-1α), fatty acid oxidation (CPT-1, COX-1), nor the marker for mitochondrial numbers in cells (VDAC) [31] were altered by HFru or HFat feeding (Figure 4C). However, the activity of citrate synthase, an enzyme that catalyses the first step in the citric acid cycle, was significantly enhanced by both HFru and HFat feeding (both *p*<0.05 vs CH; Figure 4E). HFat feeding resulted in an increase activity of β-HAD (*p*<0.05 vs CH), while the effect of HFru feeding was not statistically significant (*p*>0.05 vs CH; Figure 4F). These results indicate that mitochondrial function was up-regulated in both HFat and HFru feeding, suggesting increased rather than decreased oxidation of fatty acids.

### Neither HFru nor HFat feeding was associated with oxidative stress in the liver

**Figure 5:**
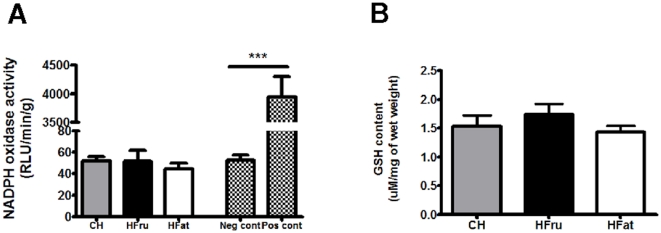
Effects of HFru and HFat feeding on oxidative stress. After one week of HFru or HFat feeding, the activity of NADPH oxidase (**A**) and intracellular GSH content (**B**) were quantified in liver homogenates, data are mean ± SE of 6 to 8 mice per group. *** *p*<0.001.

As reactive oxygen species are important by-products of an increased β-oxidation of long chain fatty acids which could cause oxidative stress to the tissue, we measured NADPH oxidase activity and glutathione (GSH) content for evidence of oxidative stress. In contrast to the increased activity of β-HAD, HFat feeding did not result in an increase activity of NAHPH oxidase (Figure 5A) or a reduction in whole tissue GSH content (Figure 5B) which are hallmarks of oxidative stress [32], [33].

### HFru and HFat feeding exerted different effects on JNK, IKK activation and ER stress

**Figure 6:**
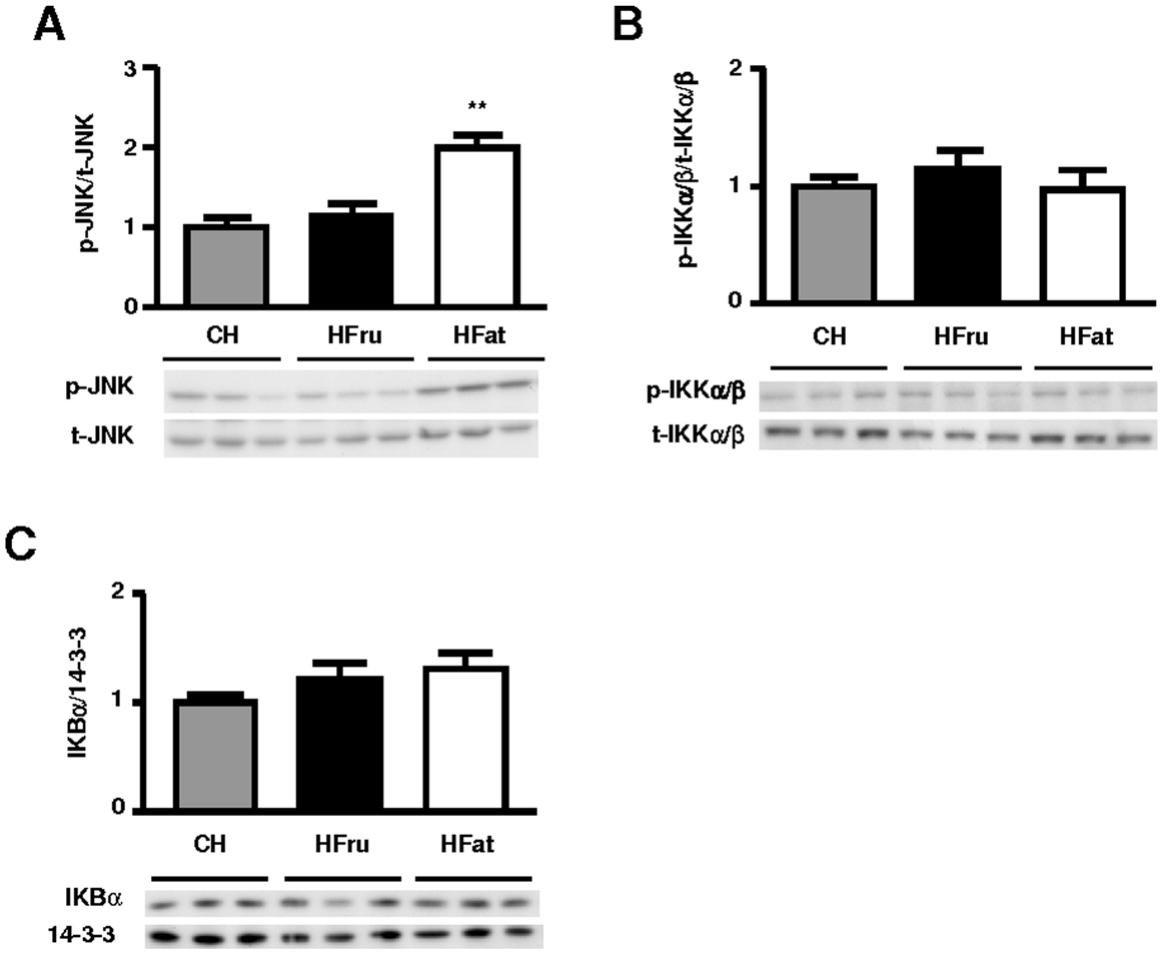
Effects of HFru and HFat on JNK and IKK activation. After one week of HFru or HFat feeding, liver homogenates were immunoblotted for p-JNK/t-JNK (**A**), and p-IKKα/β/t-IKKα/β (**B**) and t-IKBα (**C**). Each lane represents a single mouse. Densitometry data are mean ± SE of 6 mice per group. ** *p*<0.01 vs CH fed mice.

Activation of JNK and IKK has been suggested to be associated with hepatic steatosis and impaired insulin signal transduction [15]. We found a 2 fold increase in p-JNK/t-JNK in HFat-fed mice (*p*<0.01; Figure 6A), indicating the presence of cellular stress in response to extrahepatic lipid oversupply. In HFru-fed mice, however, the phosphorylation of JNK remained unaltered. The relative phosphorylation of IKK α/β and the total levels of IκBα in both HFru and HFat-fed mice were not significantly different from CH-fed mice (Figure 6B & C).

**Figure 7:**
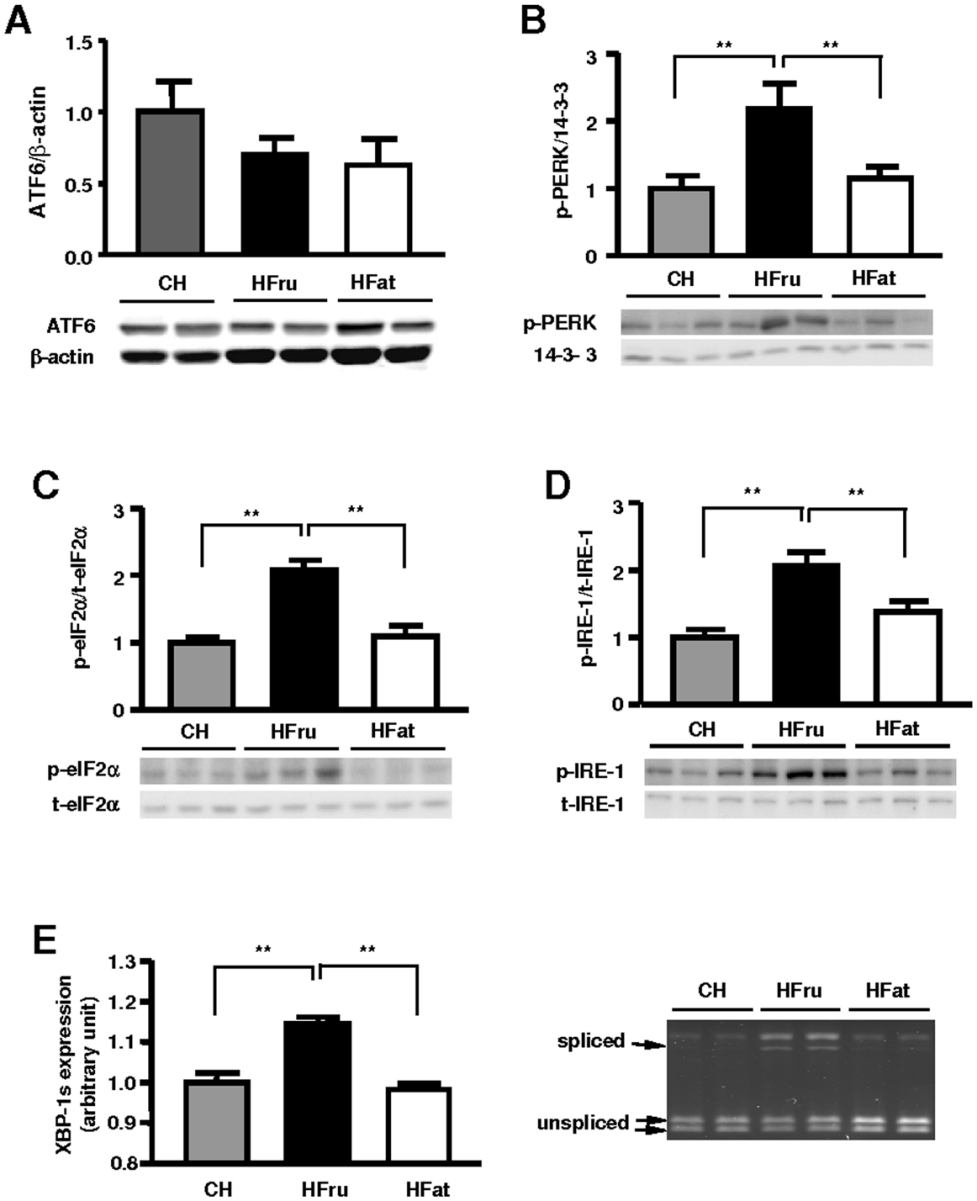
Effects of HFru and HFat on ER stress markers in the liver. After one week of HFru or HFat feeding, animals were fasted for 5-7 hours before tissue collection and liver homogenates were immunoblotted for markers of ER stress. (**A**) ATF6/β-actin (n = 4/group), (**B**) p-PERK, (**C**) p-eIF2α/t-eIF2α, (**D**) p-IRE1/t-IRE1, and (**E**) the post-transcriptional splicing of XBP1 transcript. Each lane represents a single mouse. Densitometry data are mean ± SE of 6 mice per group. ** *p*<0.01.

Recent studies have highlighted ER stress as an important mechanism integrating various pathways leading to insulin resistance during obesity [15], [16]. To investigate whether ER stress was associated with the pathogenesis of hepatic steatosis, we examined the activation of the unfolded protein response (UPR) pathways for markers of ER stress. The protein level of ATF6 was not affected by either HFru or HFat diet (Figure 7A). However, HFru feeding markedly increased the phosphorylation of PERK (2.2 fold), eIF-2α (2.6 fold) and IRE1 (2.1 fold; *p*<0.01; Figure 7B–D). Consistent with the increased IRE1 phosphorylation, the abundance of the spliced form of the X-box binding protein 1 (XBP1s) mRNA was also increased (Figure 7E). However, no significant changes of these markers were detected in HFat-fed mice. These different effects of HFru and HFat feeding on these two ER stress pathways were also observed on day 3 and week 8 (data not shown).

## Discussion

The present study investigated the roles of mitochondrial metabolism and ER stress in the development of hepatic steatosis and insulin resistance induced by excessive DNL (HFru feeding) compared to extrahepatic lipid oversupply (HFat feeding). Our data showed that both conditions led to a rapid development of hepatic steatosis and insulin resistance without any detectable mitochondrial dysfunction. Compared with HFat diet, HFru feeding resulted in greater hepatic steatosis throughout the period of the study (8 weeks). Interestingly, DNL-induced steatosis and insulin resistance co-existed with a marked activation of the PERK/eIF2α and IRE1/XBP1 arms of the ER stress pathways while lipid oversupply was associated with the activation of JNK rather than ER stress at the early stage. In ob/ob mice, the relief of ER stress by overexpressing the chaperone protein GRP78 has been shown to reduce hepatic steatosis by inhibiting SREBP-1c mediated lipogenesis [29]. In keeping with this report, our data suggest that activation of ER stress pathways may play an important role in DNL and subsequent changes in lipid hepatic steatosis and insulin resistance during HFru feeding. Importantly, our data clearly show a divergence in ER stress pathways between intrahepatic DNL and extrahepatic lipid supply on the initiation of hepatic steatosis and insulin resistance independent of obvious mitochondrial defects. Such diverged ER stress response to DNL and lipid oversupply has not been reported in previous studies using genetically obese animals [13], [15], [16], [18].

Fructose and fat are the major dietary factors leading to the development of hepatic steatosis and insulin resistance in humans [4], [5], [7]. Chronic feeding of diets high in either of them in animals is known to cause hepatic steatosis, insulin resistance and obesity resembling the metabolic syndrome in humans [7], [8], [34]. We first examined the temporal changes of hepatic steatosis during HFru and HFat feeding. The results showed both diets generated hepatic steatosis within 3 days and this metabolic phenotype was sustained beyond one week. We have noted that HFru- and HFat-feeding resulted in different degrees of hepatic TG accumulation. This may complicate the interpretation of our findings in the HFru animals because the deleterious effects of hepatic lipid accumulation are well documented [35]. Despite the similar TG levels in the liver of the 3 day HFat-fed mice to 1–8 weeks of HFru-fed mice, the HFat-fed mice did not exhibit the same ER stress markers. These data indicate that the observed hepatic ER stress induced by HFru feeding is unlikely to result from the greater TG levels *per se* when compared with HFat feeding.

As expected, one week of HFru feeding substantially increased hepatic DNL along with a dramatic up-regulation of lipogenic enzymes ACC, FAS and SCD-1, mediated mainly by SREBP-1c and as previously suggested [1], [36]. Although ChREBP protein levels were similar among the three groups, we did detect a 50% increase in ChREBP mRNA with HFru feeding (*p*<0.05) as previously reported [37]. Further studies are needed to clarify the role of ChREBP in fructose-induced DNL as ChREBP protein requires nuclear translocation to exert its function [38], [39]. In contrast, HFat feeding significantly down-regulated ACC and SCD-1 and suppressed [^3^H]-H_2_O incorporation into triglyceride, confirming the inhibitory effect of dietary fat (long chain fatty acids) on hepatic DNL and the involvement of ACC and SCD-1 as previously reported [23], [40]. Since the activation of SREBP-1c is dependent on the proteolytic cleavages [36] and this process is influenced by fatty acids [41], the suppressed DNL during the HFat feeding may be due to the inhibitory effects of an increased influx of fatty acids at the sites distal from SREBP-1c.

Mitochondrial dysfunction has been shown to be associated with hepatic steatosis and insulin resistance in humans [9], [42]. Decreased fatty acid oxidation along with impaired mitochondrial function has been demonstrated in animal models with severe NAFL such as diabetic ZDF rats [43] and OLETF obese rats [11], and mice fed a high fructose corn syrup (HFCS-55) enriched diet for 30 weeks [44]. We measured markers of mitochondrial content and also substrate oxidative capacity in tissue homogenates, and found no obvious mitochondrial defects following HFru or HFat feeding. In fact, the activity of citrate synthase was enhanced by both HFru and HFat feeding with an increased β-HAD activity found in the HFat group. The lack of liver mitochondrial dysfunction has been observed in HFat-fed mice in our previous studies (4–5 wks of feeding) [23], [45] and others [46]. These findings together suggest that liver mitochondrial dysfunction is likely to be a consequence of prolonged lipid toxicity effects [46] which may exacerbate hepatic steatosis rather than a primary contributor in the early stage.

Hepatic insulin resistance in both HFat and HFru-fed rodents has been well characterized by the use of hyperinsulinemic-euglycemic clamp coupled with glucose tracers [8], [21], [34]. Having confirmed the development of hepatic insulin resistance (impaired glucose tolerance and the blunted insulin-stimulated phosphorylation of Akt and GSK3β in the liver), we next investigated the involvement of JNK and IKK as mediators of steatosis and insulin resistance. JNK and IKK are the key stress-activated kinases to disrupt insulin signal transduction by serine-phosphorylating IRS1/2 leading to insulin resistance in HFat-fed mice [15]. Consistent with these reports, we detected an enrichment of p-JNK but not IKK, along with a reduced insulin-stimulated Akt and GSK3β phosphorylation in the HFat group. However, these stress pathways were not activated in the HFru group, suggesting that neither JNK nor IKK was involved in the development of hepatic steatosis and insulin resistance induced by DNL. In addition, JNK has also been shown to be the key mediator of ER stress leading to insulin resistance during hepatic steatosis [13], [15], [18]. However, we found no indication of JNK or IKK activation in HFru-fed mice, while JNK was activated in HFat mice in the absence of ER stress. These data indicate that neither JNK nor IKK is required for the induction of hepatic insulin resistance in response to an enhanced DNL [15].

Another factor that has been described as an important mechanism in causing insulin resistance is oxidative stress [47], [48], particularly in the state of elevated fatty acid oxidation [49], [50], [51], [52], [53]. However, the lack of changes in the oxidative stress indicators in the liver (NADPH oxidase activity and GSH content) suggests that the hepatic insulin resistance induced by HFat and HFru in the present study is not attributable to oxidative stress as a major factor. Given the rapid accumulation of triglyceride in the liver in both HFru and HFat mice, the observed hepatic insulin resistance is likely to result from associated increases in lipotoxic metabolites such as diacylgerol glycerol (DAG) or ceramide. Interestingly, HFru feeding has been shown to increase ceramide in the liver of mice (but not DAG) [54], [55]. As ceramide is known to dephosphorylate AKT [6], we postulate this is a likely mechanism for the reduced hepatic pAKT in response to insulin stimulation in HFru-fed mice. As for the HFat feeding, several studies have shown significant increase of DAG in the liver [56], [57], [58]. DAG can activate lipid-sensitive PKC isoforms such as -θ and -ε [59] which phosphorylates the serine/threonine residues of IRS to interrupt insulin-mediated phosphorylations at the tyrosine sites required for the signal transductions [7]. Thus, while both HFat and HFru diets are capable of causing hepatic steatosis, the mechanisms of associated hepatic insulin resistance may not be necessarily the same at the molecular level.

Recent studies have highlighted ER stress as an important mechanism integrating various pathways leading to insulin resistance during obesity [15], [16]. Previous studies have shown the involvement of the PERK/eIF2α and IRE1/XBP1 arms of the ER stress signaling pathways in hepatic steatosis and insulin resistance [15], [16]. In agreement with this, these two arms of ER stress signaling were significantly activated in response to HFru feeding. However, we did not detect any significant changes in ATF6, p-PERK, p-IRE1, p-eIF2α., or XBP1 splicing, after one week of HFat feeding when hepatic steatosis and insulin resistance were clearly present.

As ER stress can be triggered by the overload of newly synthesised unfolded proteins, the observed ER stress in this study may well result from the marked increases in lipogenic enzymes, given that high carbohydrate has been demonstrated to up-regulate lipogenic enzymes via the actions of SREBP-1c and ChREBP [36]. On the other hand, recent data have also indicated that ER stress may be able to directly promote lipogenesis [15], [17]. Of particular interest is the IRE1/XBP1 branch, which is found to be activated during elevated DNL in the present study. XBP1 is a nuclear transcriptional factor that can bind to the promoter regions of ACC and SCD-1 to increase lipogenesis [17]. XBP1 can also directly interact with the regulatory subunit of PI3K, p85α ¨, in the insulin signaling pathway to facilitate its transcriptional activity on genes involved in lipogenesis [60], [61]. It is possible that XBP-1 may interact with SREBP-1c which serves as a crosstalk point between ER stress and lipogenesis. However, further studies are needed to dissect the causal relationship among ER stress signaling pathways, lipogenesis and insulin resistance, as additional players in ER stress might be involved [15].

In summary, the present study showed that the involvement of ER stress pathways in the development of hepatic steatosis and insulin resistance is induced only by dietary HFru but not by HFat feeding, suggesting that ER stress is involved in DNL *per se* rather than resulting from hepatic steatosis. Neither JNK nor IKK is required for the ER stress-induced insulin resistance as previously suggested in genetic models of obesity [13], [15], [16], [18]. Our data also indicated that mitochondrial dysfunction is unlikely to be a primary cause of hepatic steatosis and insulin resistance induced by HFru or HFat feeding. As insulin resistance is known to be multifactorial, our studies in these two nutritional models provide new insight into the potential role of different lipid metabolic pathways linking to hepatic steatosis and insulin resistance.

## Supporting Information

Figure S1 **mRNA expression of SREBP-1c and ChREBP.** After one week of HFru or HFat feeding, mRNA expression of the lipogenic transcription factors SREBP-1c (**A**) and ChREBP (**B**) was determined by RT-PCR. Data are mean fold change ± SE relative to CH-fed mice of 6 mice per group. * *p*<0.05, ** *p*<0.01 vs CH.
(TIF)

Table S1 **Specific primers for real time PCR.**
(TIF)
