# Supplementary figures and images for: Differing Endoplasmic Reticulum Stress Response to Excess Lipogenesis versus Lipid Oversupply in Relation to Hepatic Steatosis and Insulin Resistance
Source: PLoS One. 2012 Feb 15;7(2):e30816. doi: 10.1371/journal.pone.0030816 (PMC3280252; doi:10.1371/journal.pone.0030816)

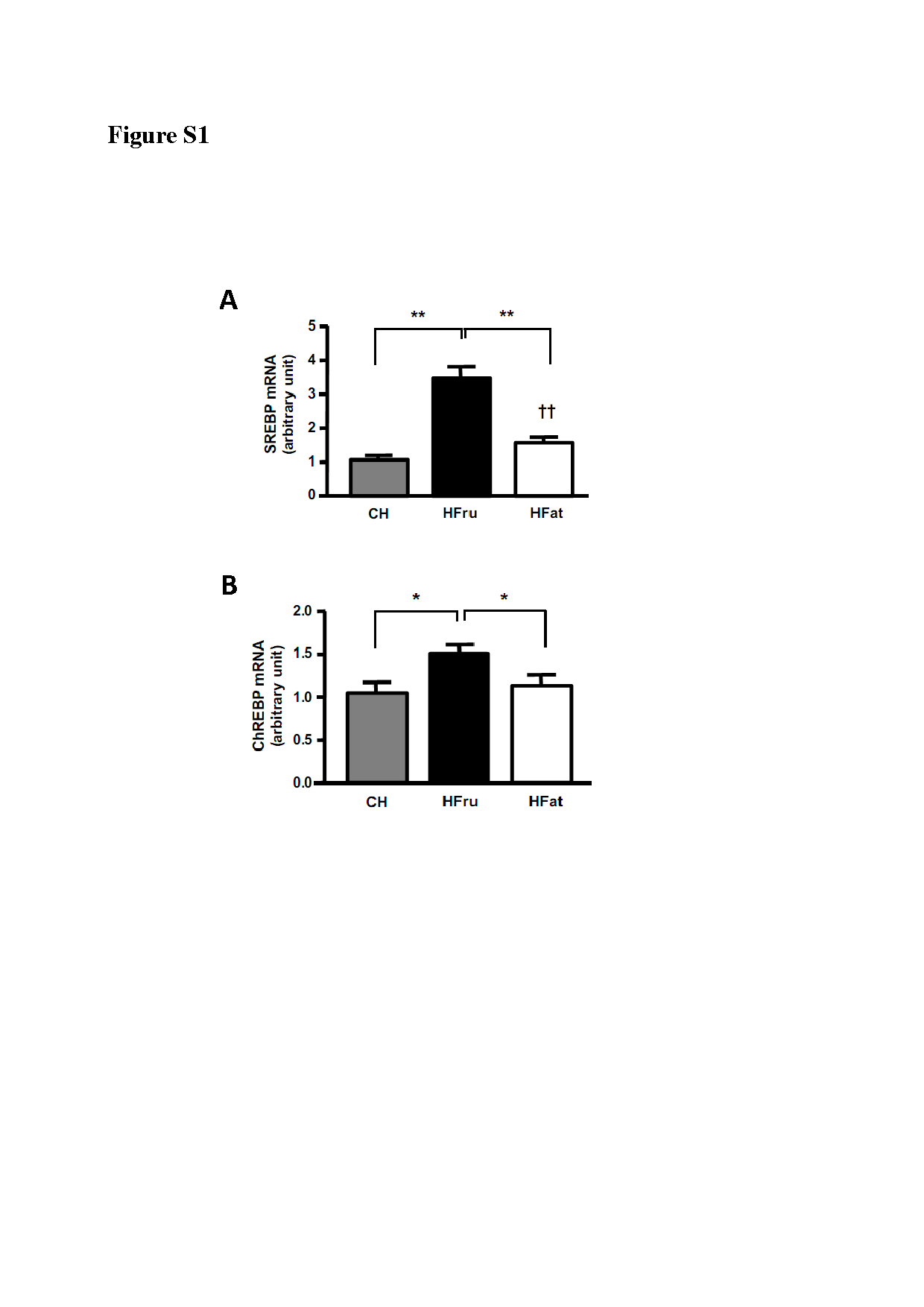

Supplement: Figure S1 — mRNA expression of SREBP-1c and ChREBP. After one week of HFru or HFat feeding, mRNA expression of the lipogenic transcription factors SREBP-1c (A) and ChREBP (B) was determined by RT-PCR. Data are mean fold change ± SE relative to CH-fed mice of 6 mice per group. * p<0.05, ** p<0.01 vs CH. (TIF) [file pone.0030816.s001.tif]

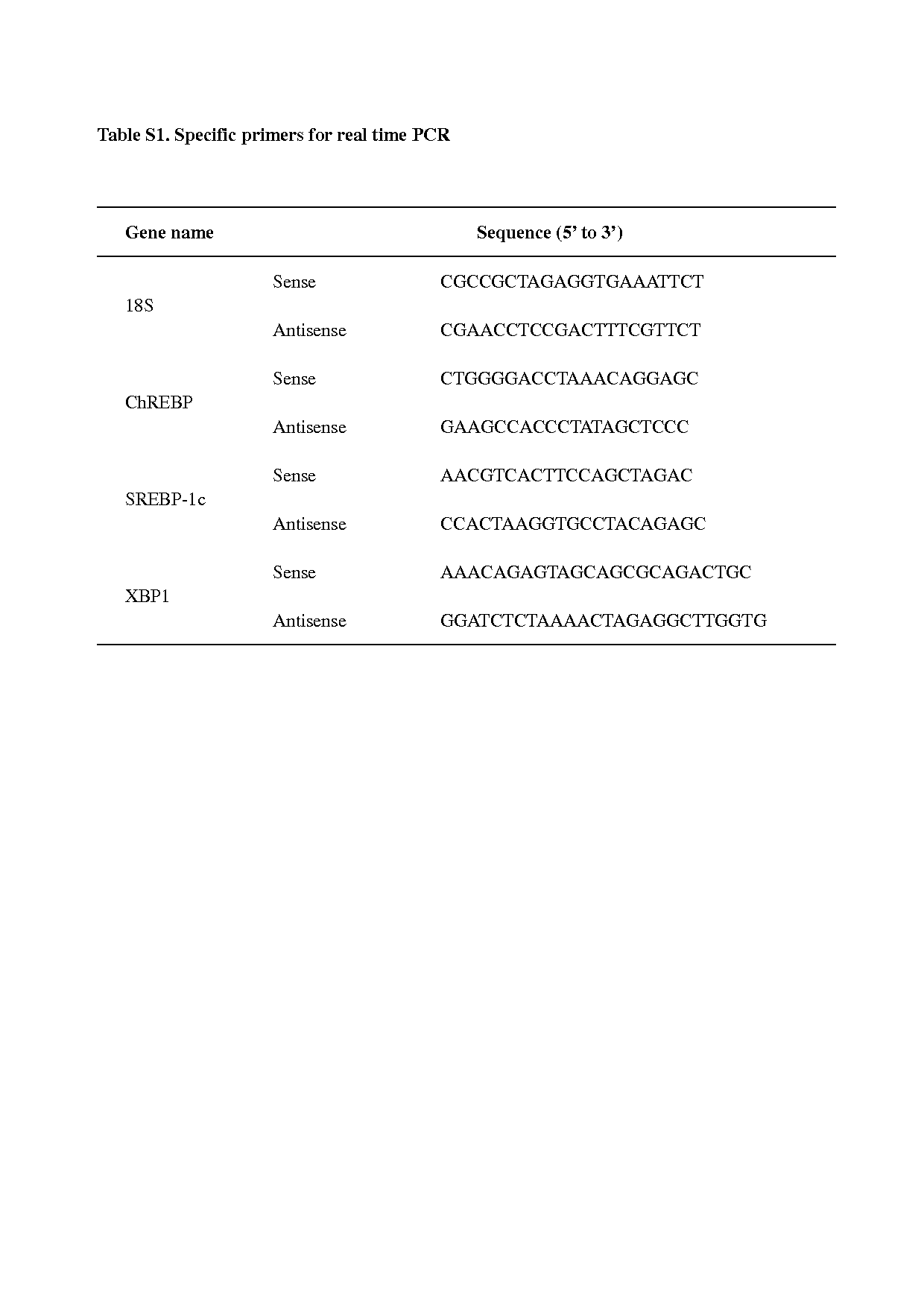

Supplement: Table S1 — Specific primers for real time PCR. (TIF) [file pone.0030816.s002.tif]
